# Erratum: Single-Cell Protein Secretomic Signatures as Potential Correlates to Tumor Cell Lineage Evolution and Cell–Cell Interaction

**DOI:** 10.3389/fonc.2013.00078

**Published:** 2013-04-08

**Authors:** Minsuk Kwak, Luye Mu, Yao Lu, Jonathan J. Chen, Yu Wu, Kara Brower, Rong Fan

**Affiliations:** ^1^Department of Biomedical Engineering, Yale UniversityNew Haven, CT, USA; ^2^Department of Electrical Engineering, Yale UniversityNew Haven, CT, USA; ^3^Isoplexis Inc.New Haven, CT, USA; ^4^Yale Comprehensive Cancer CenterNew Haven, CT, USA

We noticed an error that the name of a key contributor, Dr. Yu Wu, was left out from the author list. The corrected author list and the affiliations are the following.

Minsuk Kwak^1^, Luye Mu^2^, Yao Lu^1^, Jonathan J. Chen^1^, Yu Wu^1^, Kara Brower^1^^,^^3^ and Rong Fan^1^^,^^4^*

^1^Department of Biomedical Engineering, Yale University, New Haven, CT, USA

^2^Department of Electrical Engineering, Yale University, New Haven, CT, USA

^3^Isoplexis Inc., New Haven, CT, USA

^4^Yale Comprehensive Cancer Center, New Haven, CT, USA

